# Postnatal Loss of Hap1 Reduces Hippocampal Neurogenesis and Causes Adult Depressive-Like Behavior in Mice

**DOI:** 10.1371/journal.pgen.1005175

**Published:** 2015-04-15

**Authors:** Jianxing Xiang, Sen Yan, Shi-Hua Li, Xiao-Jiang Li

**Affiliations:** 1 Department of Human Genetics, Emory University School of Medicine, Atlanta, Georgia, United States of America; 2 State Key Laboratory of Molecular Developmental Biology, Institute of Genetics and Developmental Biology, Chinese Academy of Sciences, Beijing, China; King's College London, UNITED KINGDOM

## Abstract

Depression is a serious mental disorder that affects a person’s mood, thoughts, behavior, physical health, and life in general. Despite our continuous efforts to understand the disease, the etiology of depressive behavior remains perplexing. Recently, aberrant early life or postnatal neurogenesis has been linked to adult depressive behavior; however, genetic evidence for this is still lacking. Here we genetically depleted the expression of huntingtin-associated protein 1 (Hap1) in mice at various ages or in selective brain regions. Depletion of Hap1 in the early postnatal period, but not later life, led to a depressive-like phenotype when the mice reached adulthood. Deletion of *Hap1* in adult mice rendered the mice more susceptible to stress-induced depressive-like behavior. Furthermore, early Hap1 depletion impaired postnatal neurogenesis in the dentate gyrus (DG) of the hippocampus and reduced the level of c-kit, a protein expressed in neuroproliferative zones of the rodent brain and that is stabilized by Hap1. Importantly, stereotaxically injected adeno-associated virus (AAV) that directs the expression of c-kit in the hippocampus promoted postnatal hippocampal neurogenesis and ameliorated the depressive-like phenotype in conditional *Hap1* KO mice, indicating a link between postnatal-born hippocampal neurons and adult depression. Our results demonstrate critical roles for Hap1 and c-kit in postnatal neurogenesis and adult depressive behavior, and also suggest that genetic variations affecting postnatal neurogenesis may lead to adult depression.

## Author Summary

Although the majority of the neurons in the brain are generated during embryonic stage, new neurons are continuously being produced postnatally, and at a much lower rate in adulthood. As postnatal neurogenesis is a key component of the brain maturation process that creates dynamic ‘wirings’ in the brain necessary for an individual to grow, learn, and cope with the external world, attenuated postnatal neurogenesis may affect an individual’s mental stability, rendering a higher susceptibility to depression later in life. In the current study, we genetically ablated the expression of huntingtin-associated protein 1 (Hap1) in mice at various ages or in selective brain regions, and found that early loss of Hap1 significantly reduces postnatal hippocampal neurogenesis, and leads to adult depressive-like behavior. We also found c-kit as an effector to mediate the neurogenesis defect and adult depressive-like phenotype in mice lacking Hap1. The results provide the first genetic evidence to demonstrate the importance of postnatal neurogenesis in adult depression, and may offer new avenues in the prevention and treatment of depression. Our study also has potential implications to other adult-onset mental disorders.

## Introduction

Depression is the most common mental disorder and a leading cause of disability around the world [1, 2]. In the US, the lifetime prevalence for major depression is estimated to be as high as 16.2% [3]. There are a variety of symptoms associated with depression, including anhedonia, depressed mood, fatigue, helplessness, and other cognitive and metabolic abnormalities [4, 5]. Despite its wide influence, the causes of depression have not been made clear, nor have we established effective and long-lasting treatments for it. To gain insight into its etiology, twin studies were conducted to determine whether genetics could play a role in depression. The results revealed that genetic factors account for about 40% of the risk of developing depression, with the remaining 60% being due to environmental factors [6].

Despite various genetic and environmental causes of depression, there must be some common pathways that lead to depressive symptoms. It has been long proposed that deficiency in serotonin (5-HT) level may underlie depression as selective serotonin reuptake inhibitors (SSRIs), the most frequently used antidepressant drugs, work through enhancing extracellular levels of 5-HT [7, 8]. Furthermore, synaptic dysfunction [9, 10], hyperactivity of the hypothalamic-pituitary-adrenal (HPA) axis [11], and expression changes or polymorphisms of brain-derived neurotrophic factor (BDNF) may also be associated with depression [12]. Recently, hippocampal neurogenesis has emerged as an attractive theory of depression [13, 14], largely because many antidepressants are known to enhance hippocampal neurogenesis [15–18], and ablating adult neurogenesis reduces some of the behavioral effects of antidepressants [19]. Of note, many of the common theories of depression such as those aforementioned have also been closely linked to adult neurogenesis [20–23], further accentuating its importance in depressive behavior. Although much of the focus in depression research has been on adult neurogenesis, postnatal neurogenesis, which occurs early in life, is also important as an increasing number of studies found that it is capable of influencing adult depressive behavior [24–27]. Nevertheless, most if not all of these studies used either chemicals or maternal separation to induce a decline in postnatal neurogenesis. To our knowledge, a genetic model for investigating the relationship between postnatal neurogenesis and adult depression is still lacking.

Huntingtin-associated protein a (Hap1) is an intriguing neuronal-enriched protein that interacts with several disease-related proteins, including huntingtin and Ahi1, whose mutations cause Huntington's disease (HD) and Joubert syndrome, respectively [28, 29]. Mounting evidence shows that Hap1 mediates the intracellular transport of several neurotrophic factors and their receptors to support neuronal function and survival, which may require a concerted effort with huntingtin [30–37]. The involvement of Hap1 in BDNF/TrkB trafficking [33–35] may be especially important as BDNF is the most abundant neurotrophic factor in the nervous system and is essential for neuronal activity and the survival of animals. Our recent work revealed that Hap1 regulates early postnatal hypothalamic neurogenesis by stabilizing BDNF/TrkB signaling and that this hypothalamic neurogenesis is critical for the postnatal growth and survival of mice [32]. Earlier work found that neuronal deficiency of the Hap1-binding protein Ahi1 leads to a depressive-like phenotype in adult mice [38]. In the current study, we found that selective depletion of Hap1 expression at postnatal ages also led to adult depressive-like behavior, which was associated with reduced postnatal neurogenesis in the hippocampus. Moreover, we discovered that this decreased neurogenesis is mediated by a different mechanism involving c-kit, a receptor for stem cell factor (SCF) that is also expressed in neural progenitor or stem cells in the rodent brain [39–41]. c-kit is downregulated in *Hap1* KO mouse hippocampus because its stability requires Hap1. Our results also demonstrate that overexpression of c-kit in the postnatal hippocampus can augment hippocampal neurogenesis and alleviate adult depression, suggesting a new mechanism by which Hap1 regulates postnatal neurogenesis and consequent adult depressive behavior.

## Results

### Early Postnatal Depletion of Hap1 Leads to Depressive-Like Behavior in Adult Mice

**Fig 1:**
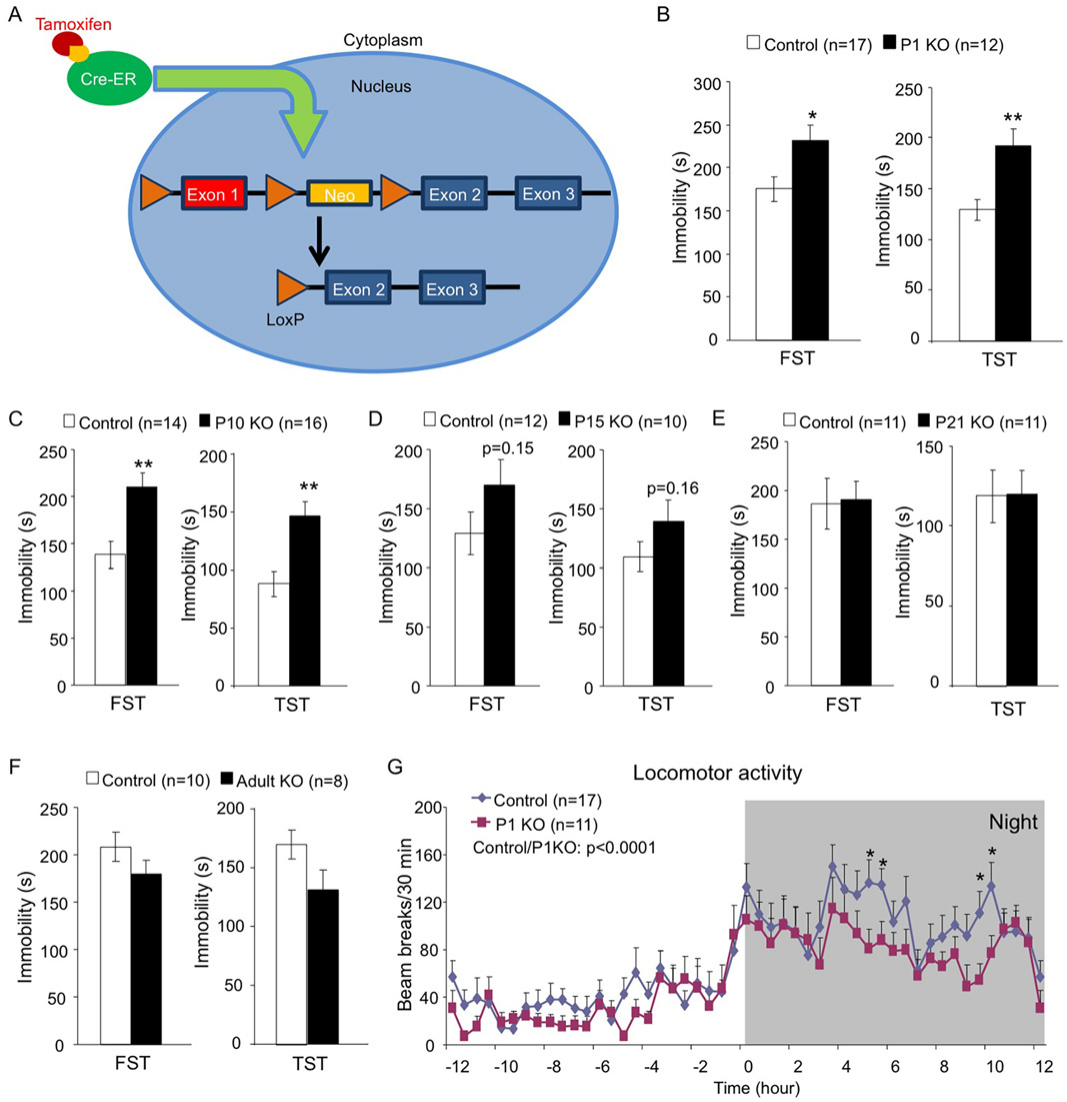
Early postnatal depletion of Hap1 leads to depressive-like behavior in adult mice. (**A**) A schematic of the TM-inducible Cre/loxP system to delete *Hap1* in mice. (**B-F**) Forced swim and tail suspension tests (FST and TST) were performed at 3 months of age for *Hap1* P1 (B, P1 KO), P10 (C, P10 KO), P15 (D, P15 KO) and P21 (E, P21 KO) KO mice, and 4 months of age for mice that had *Hap1* gene deleted at 2 months old (F, adult KO). Note that early postnatal Hap1 depletion, but not late postnatal or adult depletion, led to increased immobility in the FST and TST. (**G**) Locomotor activities of 2-month-old *Hap1* P1 KO and control mice were monitored and recorded for 24 h. The KO mice showed a general reduction in locomotor activity. All error bars represent SEM. *p<0.05, **p<0.01. In (G), two-way (genotype and time) ANOVA and post hoc tests were performed to determine statistical significance.

Since mice with neuronal deficiency of the Hap1-interacting protein Ahi1 show depressive phenotypes [38], we were interested to see whether *Hap1* KO mice would display similar phenotypes and whether *Hap1* deletion at different ages would affect behavioral outcomes. To test these questions, we used the Cre-LoxP system via tamoxifen (TM) induction to induce *Hap1* deletion at different postnatal stages (Fig 1A). Substantial depletion of Hap1 protein expression was achieved in all KO groups as analyzed by western blot (S1 Fig). Two to three months after the TM induction, we subjected the Hap1-depleted mice to the forced swim test (FST) and tail suspension test (TST). These tests showed that early postnatal (Fig 1B and 1C), but not late postnatal (Fig 1D and 1E) or adult (Fig 1F), depletion of Hap1 resulted in significant increases in immobility, which is considered depressive-like behavior. Since the relationship between decreased or altered locomotion and depression has been suggested by previous clinical studies [42–45], we also tested locomotor activity in these mice and found that depletion of Hap1 at an earlier age also resulted in lower locomotor activity in adult mice compared to controls (Fig 1G).

**Fig 2:**
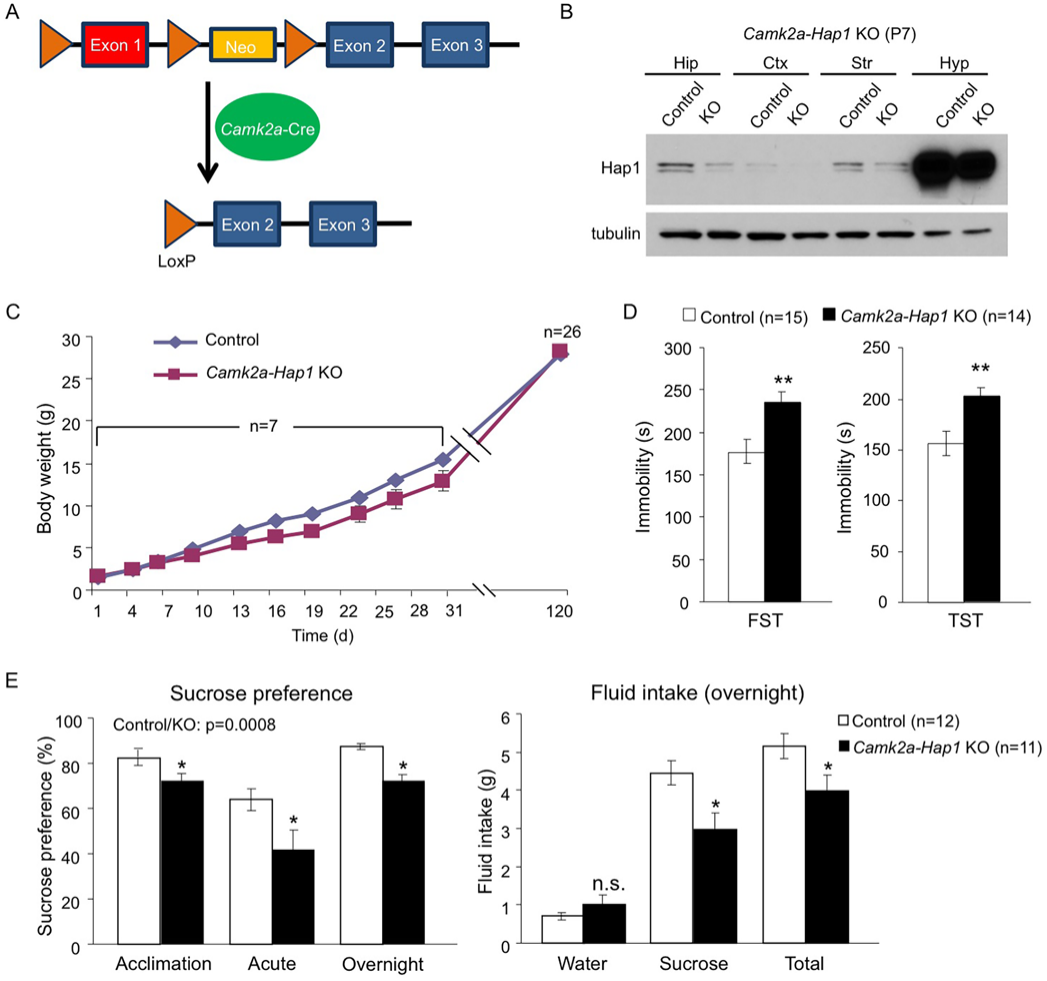
*camk2a-Hap1* conditional KO mice also display adult depressive-like behavior. (**A**) A schematic of conditional *Hap1* KO in *camk2a*-Cre-expressing neurons in mice. (**B**) Western blotting result showing that the Hap1 protein level is decreased in the forebrain of *camk2a-Hap1* KO mice at P7. Note that the decrease in the hippocampus is much more dramatic than in the hypothalamus. Hip: hippocampus; Ctx: cortex; Str: striatum; Hyp: hypothalamus. (**C**) Body weight recording of *camk2a-Hap1* KO and control mice. Daily body weight recording was done for the first month using 2 litters of mice. At 4 months of age, 7 litters were used. (**D**) FST and TST revealed depressive-like behavior in 2-month old *camk2a-Hap1* KO mice. (**E**) Sucrose preference was assessed during acclimation and in acute and overnight tests. Two-month-old *camk2a-Hap1* KO mice showed reduced preference compared to controls. Fluid intake was also measured for the overnight test. *Camk2a-Hap1* KO mice consumed significantly less sucrose solution than controls. All error bars represent SEM. n.s., not significant, *p<0.05, **p<0.01.

To further strengthen the idea that early postnatal Hap1 depletion leads to an adult depressive phenotype, we crossed floxed-*Hap1* mice with *camk2a*-Cre transgenic mice and obtained *Hap1* conditional KO mice in which *Hap1* is deleted in *camk2a*-expressing neurons (Fig 2A). Based on *camk2a*-promoter activities, Hap1 expression was diminished early postnatally to varying extents in the forebrain areas, with the greatest reduction seen in the cortex and hippocampus (Fig 2B). As *camk2a-Hap1* KO mice still maintained a fairly high level of hypothalamic Hap1 expression, which controls feeding and growth [46], we only saw a mild decrease in the body weight gain of these mice at the postnatal stage (Fig 2C). The KO mice displayed a mild decrease in body weight during postnatal life, but the body weight difference between KO and control mice at 4 months old was indiscernible. To see whether *camk2a-Hap1* KO mice would behave similarly in the depression tests as the induced *Hap1* KO mice, we performed the FST and TST, which demonstrated that *camk2a-Hap1* KO mice at 2-month old indeed displayed depressive-like behavior (Fig 2D).

To rule out the possibility that our *Hap1* KO mice had any physical impairment that might result in poor performances in depression tests, as well as a locomotor activity assay, we did a rotarod test on both P1 KO and *camk2a-Hap1* KO mice; we found no such impairment in either of the KO groups (S2A Fig). Instead, P1 KO mice displayed enhanced performance, which very likely could be the result of their smaller body size allowing them to more readily balance and cling on to the rod. Imipramine, a tricyclic antidepressant, is used in the treatment of major depression and can rapidly reduce depressive phenotypes in mice [38, 47]. To examine whether this drug could rescue the depressive phenotype in Hap1-depleted mice, we delivered imipramine via intraperitoneal injection (i.p.) and found that it substantially increased the mobility of *camk2a-Hap1* KO mice in both the FST and TST, more so than it did to the control mice (S2B Fig). As a result, the mobility of the mice after treatment was not significantly different between the genotypes (S2B Fig).

To further validate the depressive phenotype, we assessed anhedonia in *camk2a-Hap1* KO mice using the sucrose preference test. Anhedonia, or an inability to experience pleasure, is considered the core feature of major depression [48]. In rodents, the sucrose preference test is used widely to assess anhedonia based on the finding that depressed human patients have higher hedonic responses to sucrose solutions than controls [49]. We found that *camk2a-Hap1* KO mice displayed lower preference to 1% sucrose solution than controls, as measurements of fluid intake indicated that, although water intake was not different between the genotypes, *camk2a-Hap1 KO* mice consumed significantly less sucrose solution and total fluid (Fig 2E). Collectively, these results support a depressive-like phenotype caused by Hap1 depletion.

### Early Postnatal Hap1 Depletion Affects Hippocampal Neurogenesis

**Fig 3:**
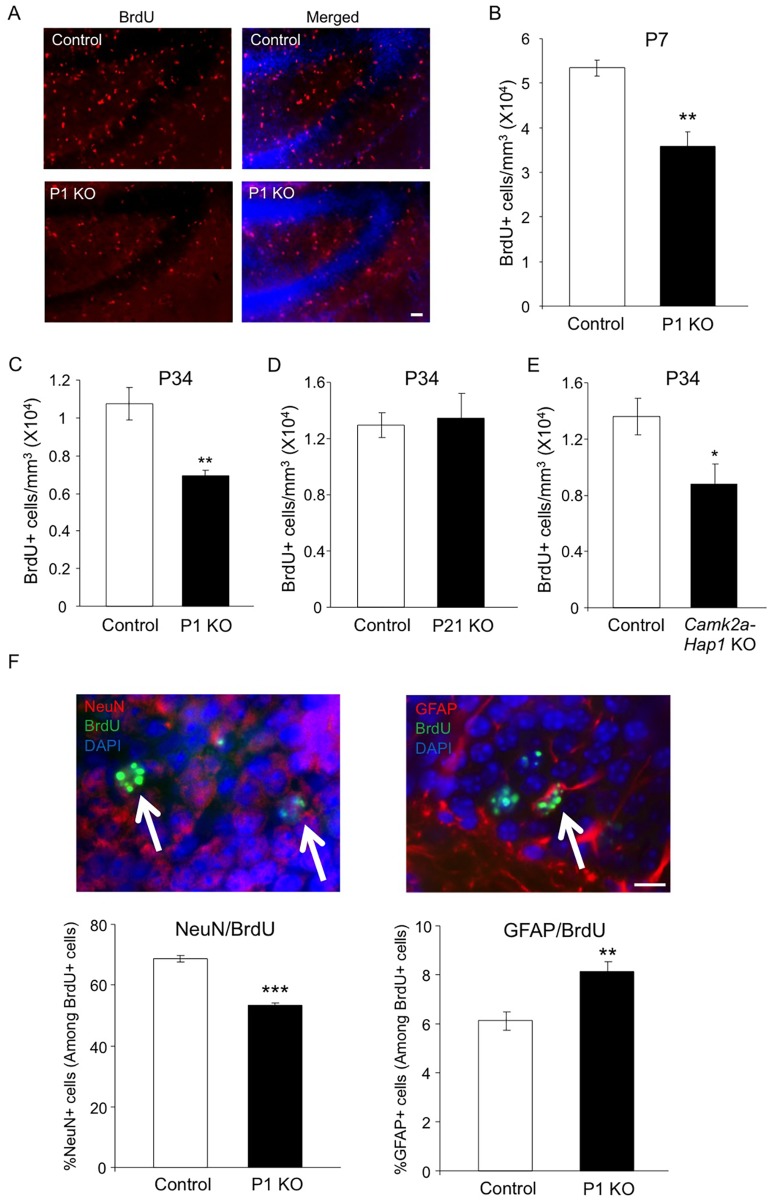
Early postnatal Hap1 depletion leads to reduced hippocampal neurogenesis. (**A**) *Hap1* P1 KO and control mice were injected with BrdU at P6, and then perfused at P7 for immunostaining of BrdU in the DG of the hippocampus. P1 KO mice showed significantly fewer BrdU+ cells in the DG. Scale bar: 40 μm. (**B**) Stereological quantification of BrdU+ cells in (A). n = 4 per genotype. (**C, D**) Stereological quantification of BrdU+ cells in the DG of *Hap1* P1 KO (C) or *Hap1* P21 KO (D) and control mice at P34 that were injected with BrdU at P33. n = 4 for control, n = 5 for KO. (**E**) Stereological quantification of BrdU+ cells in the DG of P34 *Camk2a-Hap1* KO and control mice that were injected with BrdU at P33. n = 5 for control, n = 4 for KO. (**F**) Quantification of the ratios of NeuN+ (left panel) and GFAP+ cells (right panel) among BrdU+ cells. n = 4 per genotype. Double staining images of NeuN+/BrdU+ and GFAP+/BrdU+ cells are also presented. Arrows indicate double-positive cells. Scale bar: 10 μm. All error bars represent SEM. *p<0.05, **p<0.01, ***p<0.001.

Hippocampal neurogenesis occurs abundantly after birth and remains active in adulthood. As the involvement of hippocampal neurogenesis in adult depression has been widely demonstrated [13], we next examined whether Hap1 also regulates hippocampal neurogenesis. Thus, we first injected BrdU into P6 *Hap1* P1 KO mice and controls, and analyzed BrdU incorporation 24 hours later. BrdU immunostaining and stereological quantification clearly showed a decrease in proliferating cells in the hippocampal DG (Fig 3A and 3B), indicating that early postnatal Hap1 expression may positively regulate hippocampal neurogenesis. We then performed the same assay on *Hap1* P1 KO and P21 KO mice at P34 and found that, while Hap1 depletion at P1 still had an effect on DG neurogenesis at P34, late postnatal *Hap1* KO (P21 KO) could no longer influence hippocampal neurogenesis (Fig 3C and 3D). Since *camk2a-Hap1* KO mice started to deplete Hap1 weakly at the late embryonic stage and much more potently after birth [50, 51], we went on to assess BrdU incorporation in *camk2a-Hap1* KO mice to see whether the observed adult depressive phenotype in these mice was associated with a decrease in postnatal neurogenesis. We found that *camk2a-Hap1* KO mice also had a reduced number of BrdU+ cells in the DG (Fig 3E), further supporting an important role for early postnatal Hap1 expression in hippocampal neurogenesis.

Neural progenitor cells (NPCs), when undergoing differentiation, can give rise to both neuronal and glial cells. Because of the decreased number of proliferating NPCs as indicated by the number of BrdU+ cells, we also wanted to know whether the differentiation of NPCs was perturbed by the early postnatal loss of Hap1. To explore this, we injected BrdU into *Hap1* P1 KO and control mice at P6 and sacrificed them 4 weeks later so that NPCs, which incorporated BrdU at P6, would differentiate into either mature neurons or glia. Co-immunostaining of BrdU with NeuN, a marker for mature neurons, or GFAP, a marker for mature astrocytes, revealed that P1 deletion of *Hap1* significantly affected neuronal differentiation, as the ratio of NeuN+/BrdU+ cells in P1 KO mice (53%) is significantly lower than in controls (70%) (Fig 3F, left panel). In contrast, the ratio of GFAP+/BrdU+ cells among BrdU+ cells was increased in P1 KO mice (Fig 3F, right panel). Because Hap1 expression is not seen in mature glial cells [52, 53], we believe that this increase is unlikely to be due to the direct effect of Hap1 on glial cell differentiation, but rather is a reflection of decreased neuronal differentiation in P1 KO mice.

### Adult Hap1 Expression Protects Animals against Stress-Induced Depression by Maintaining Hippocampal Neurogenesis

**Fig 4:**
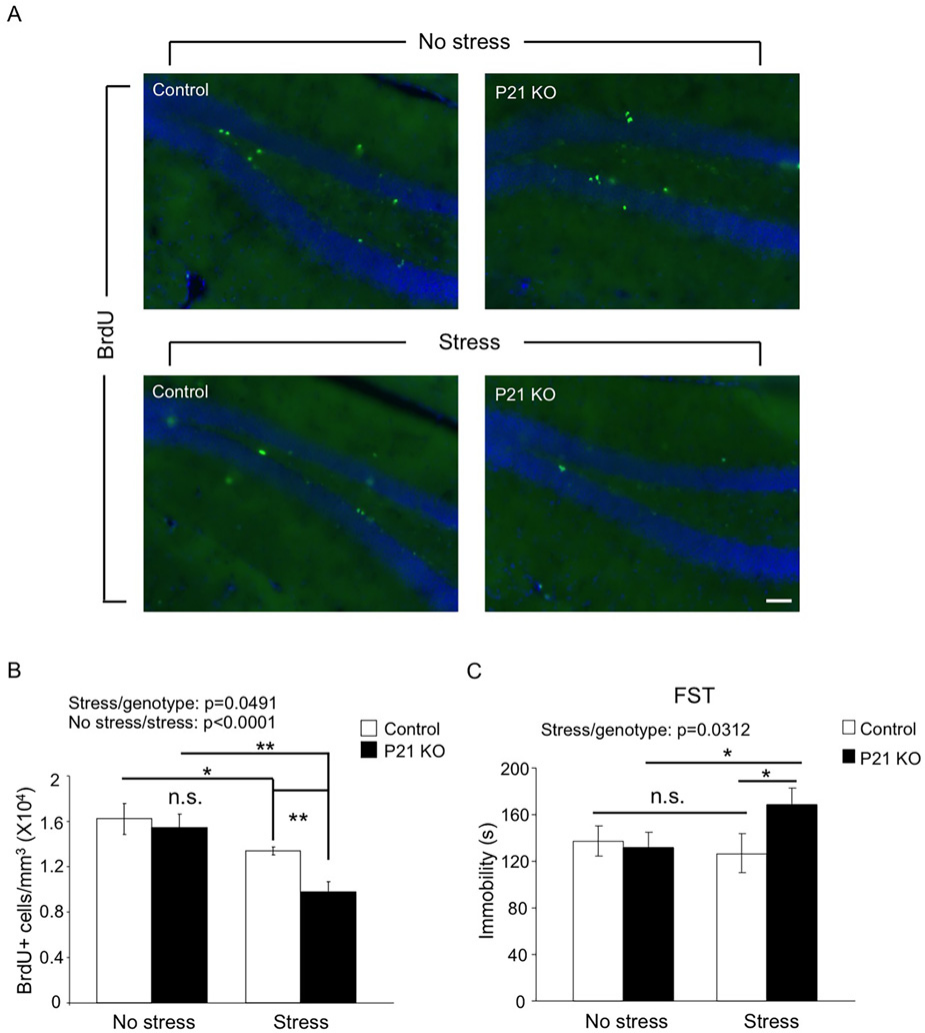
Adult Hap1 expression reduces the stress-induced defective neurogenesis in the hippocampus and depressive-like behavior. (**A**) One-year-old *Hap1* P21 KO and control mice were subjected to 4 h restraint stress for 7 consecutive days. BrdU was i.p. injected in the final 3 days of the treatment. Immunostaining of BrdU in the DG was performed 1 day after the final treatment. Scale bar: 50 μm. The stressed groups showed fewer BrdU+ cells than non-stressed groups, and the decrease was more dramatic for stressed KO mice than stressed controls. (**B**) Stereological quantification of BrdU+ cells in (A). n = 3–4 mice per group. (**C**) The forced swim test was conducted 1 day after the final treatment. n = 9–11 per group. While stressed control mice did not exhibit depressive-like behavior compared to non-stressed controls, loss of Hap1 caused stressed KO mice to display significant depressive-like behavior compared with stressed control and non-stressed KO mice. All error bars represent SEM. *p<0.05, **p<0.01.

Since neural activities and signal transduction are very different between developing and mature brains, many genes are expected to play differential roles at these two stages. Whether Hap1 plays differential roles in neurogenesis in postnatal and adult brains has gone unexplored. As the hippocampus is a brain region known to be highly responsive to stress [21], we wanted to test the idea that adult-expressed Hap1 may respond to stress in the regulation of hippocampal neurogenesis and animal behavior. To this end, we used *Hap1* P21 KO mice, which did not present gross phenotypes, including deficits in neurogenesis, and subjected them to 7-day repeated restraint stress. P21 KO mice did not exhibit impaired hippocampal neurogenesis under normal conditions; however, after repeated stress, they showed a significant reduction in hippocampal neurogenesis, much greater than in control mice (Fig 4A and 4B). Repeated restraint stress is well known to suppress hippocampal neurogenesis, but does not lead to apparent depressive behavior in WT rodents [54–57]. We thought that a certain level of neurogenesis must be maintained in order for animals to battle against the potential behavioral changes caused by stress. Because the lack of Hap1 caused hippocampal neurogenesis to drop to an abnormally low level following stress, it is possible that this reduction might undermine the ability of animals to cope with stress. Using the forced swim test (FST), we found that after repeated restraint stress, *Hap1* P21 KO mice displayed a marked increase in immobility compared to non-stressed *Hap1* KO and stressed control mice (Fig 4C), suggesting that adult expression of Hap1 is involved in the maintenance of hippocampal neurogenesis in response to certain types of stress, including restraint stress, thereby protecting animals against stress-induced depressive-like behavior.

### c-kit Levels Are Decreased in *Hap1* KO Mouse Hippocampus

**Fig 5:**
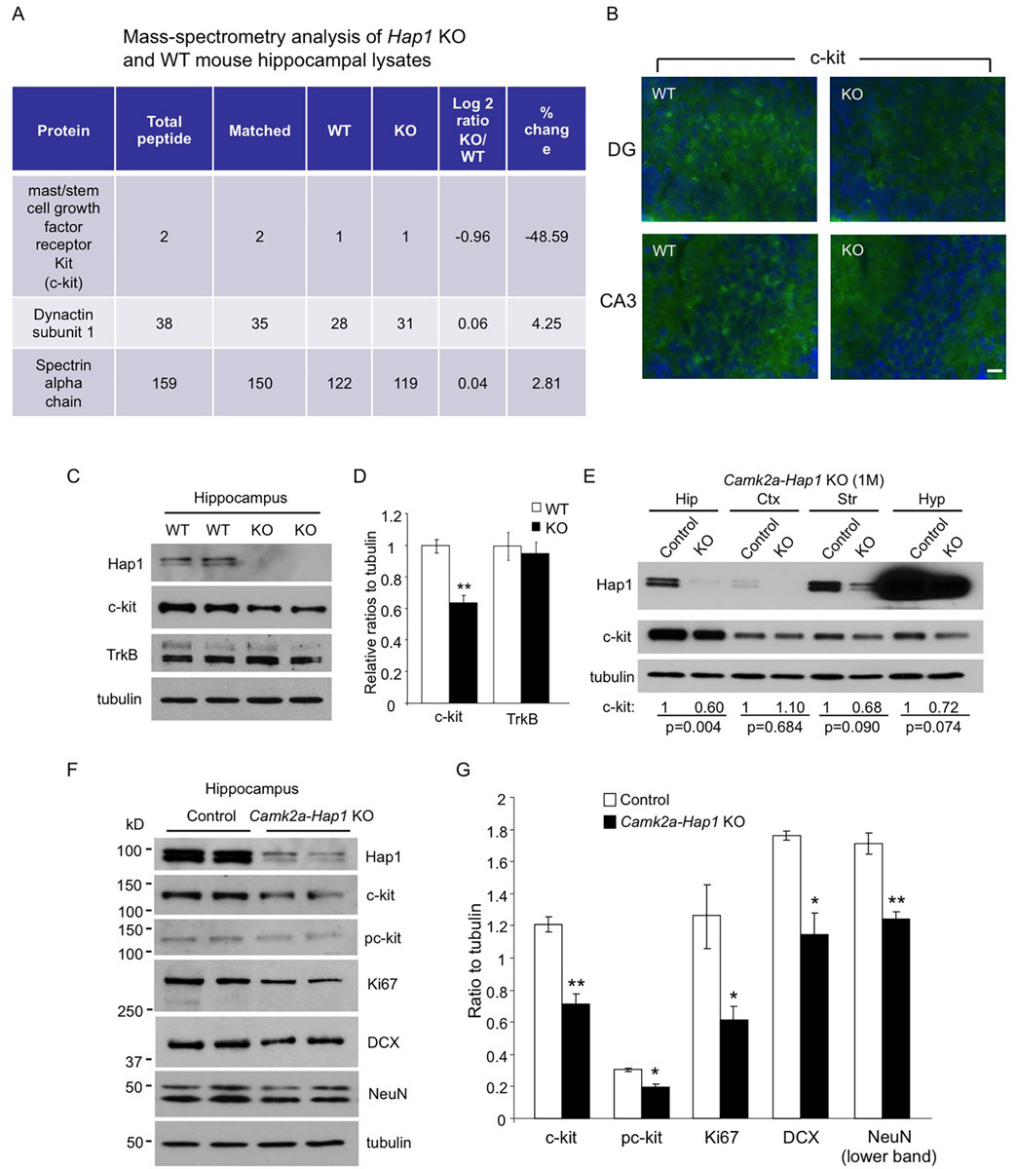
c-kit expression level is reduced in *Hap1* KO hippocampus. (**A**) Mass spectrometry analysis of P1 WT and *Hap1* KO mouse hippocampal lysates revealing reduced c-kit expression level in *Hap1* KO hippocampus. Log2 ratios were calculated based on the extracted ion current of the peptide signals from the chromatogram. (**B**) Immunofluorescent staining of c-kit on P1 WT and *Hap1* KO mouse brain sections showed decreased c-kit level in *Hap1* KO hippocampus. Scale bar: 10 μm. (**C**) Western blot analysis on WT and *Hap1* KO hippocampal lysates also confirmed the decreased c-kit protein level in KO samples. The level of TrkB, however, was not decreased. (**D**) Quantification of relative ratios of c-kit and TrkB normalized to WT is shown. n = 4 per genotype. (**E**) Western blot analysis of Hap1 and c-kit expression levels on multiple brain area lysates from 1-month-old *camk2a-Hap1* KO and control mice. Relative c-kit/tubulin ratios (normalized to control) from 3 independent experiments are shown, and the p values are also presented. (**F**) Western blot analysis of c-kit, pc-kit, and cell type-specific markers in hippocampal lysates from 1-month-old *camk2a-Hap1* KO and control mice supports attenuated c-kit signaling and neurogenesis in the KO mouse hippocampus. (**G**) Quantification of the ratios of proteins to tubulin on western blots in (F) is shown. All error bars represent SEM. *p<0.05, **p<0.01.

Our results indicate that early postnatal *Hap1* deletion reduces hippocampal neurogenesis and leads to an adult depressive-like phenotype. To find a molecular target for Hap1-regulated hippocampal neurogenesis, we prepared P1 WT and *Hap1* KO mouse hippocampal lysates and performed mass spectrometry analysis to look for potential targets of Hap1 whose levels were significantly altered between WT and KO samples. Among all the proteins identified, we found that c-kit, or mast/stem cell growth factor receptor, showed nearly 50% down-regulation in *Hap1* KO hippocampus (Fig 5A). A previous study indicated that c-kit is expressed in neuroproliferative zones of the rat brain, and in vivo administration of its ligand, stem cell factor (SCF), increases neurogenesis in these regions [40]. We first confirmed the mass spectrometry result by immunofluorescent staining (Fig 5B) and western blot analysis (Fig 5C and 5D) of c-kit, which revealed that c-kit expression in *Hap1* KO hippocampus is indeed decreased. Then, we looked at c-kit levels in *Hap1* P1 KO and adult KO hippocampal tissues. As compared with controls, c-kit expression was significantly lower in P1 KO, but not adult KO, hippocampus, suggesting that c-kit down-regulation could be associated with the decreased hippocampal neurogenesis and adult depressive-like phenotype displayed in P1 KO mice (S3A and S3B Fig). Our previous results indicate that Hap1 controls postnatal hypothalamic neurogenesis by stabilizing TrkB protein level [32]. To find out whether the same mechanism also underlies Hap1-mediated regulation of postnatal hippocampal neurogenesis, we assessed TrkB levels in germline *Hap1* KO as well as P1 and adult KO hippocampal tissues, and found that neither of these KO tissues showed a reduction in TrkB level (Fig 5C, 5D, S3A and S3B Fig), suggesting that Hap1 may regulate hippocampal neurogenesis through different signaling molecules, such as c-kit. We next examined c-kit expression levels by western blot in various tissues from *camk2a-Hap1* KO and control mice. Besides the hippocampus, which showed a significant reduction in c-kit level, we also found a trend of decreased c-kit expression in the striatum and hypothalamus of *camk2a-Hap1* KO mice (Fig 5E). Notably, c-kit is highly expressed in the hippocampus compared to other tissues examined (Fig 5E), which lends credence to the finding that SCF/c-kit signaling regulates neurogenesis. To look for more evidence that c-kit activation and neurogenesis are perturbed in *camk2a-Hap1* KO mouse hippocampus, we assessed the levels of phosphorylated c-kit (pc-kit), a proliferating cell marker, ki67, a neuroblast or immature neuron marker, DCX, and a mature neuron marker, NeuN, via western blot analysis (Fig 5F and 5G). The results showed that all these proteins were significantly reduced, indicating that loss of Hap1 affects c-kit activation by down-regulating its expression level, compromising postnatal hippocampal neurogenesis in *camk2a-Hap1* KO mice.

### Hap1 Is Partially Coexpressed with c-kit in the DG and Stabilizes Its Level In Vitro

**Fig 6:**
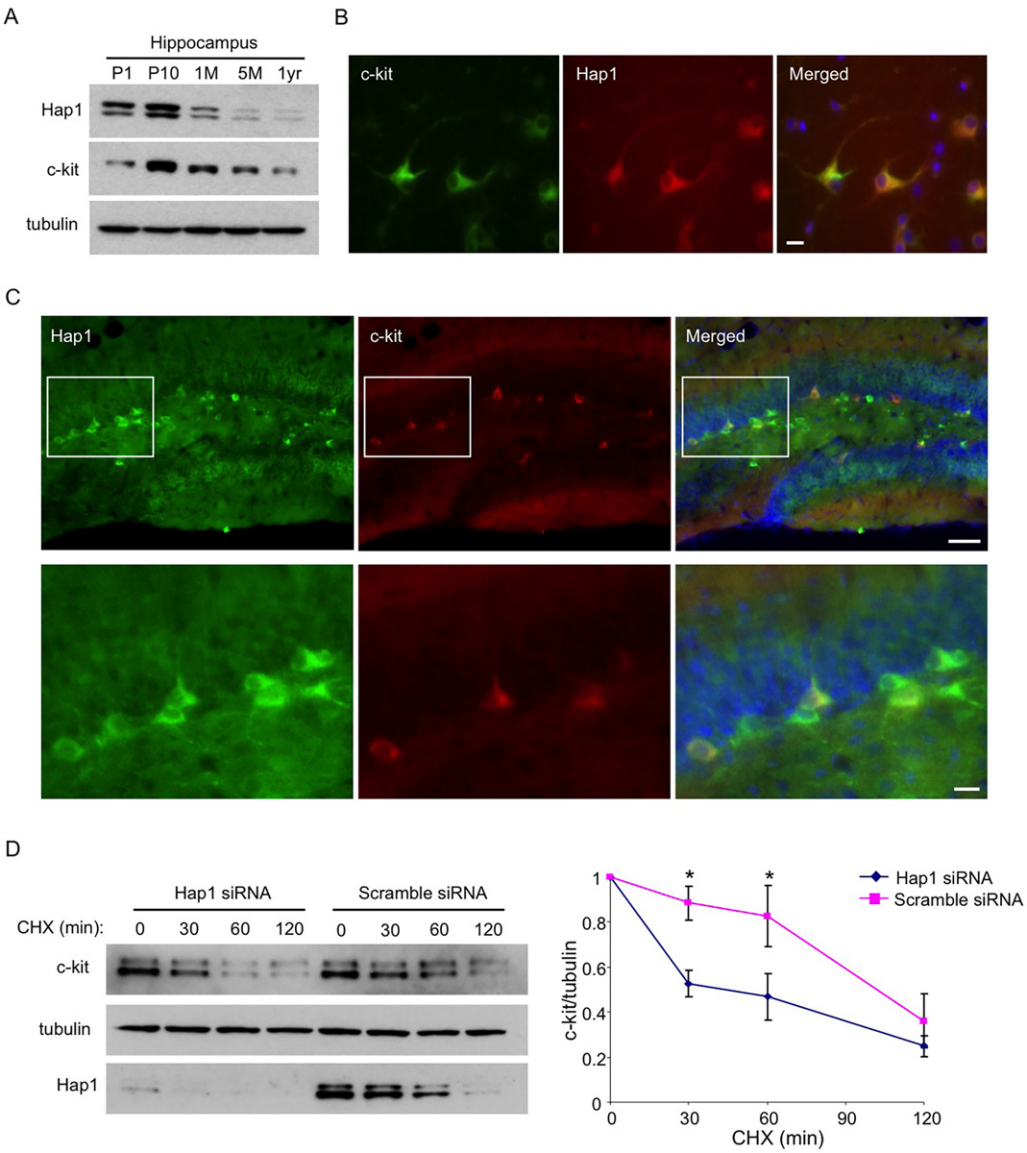
Hap1 is coexpressed with c-kit and stabilizes its level in vitro. (**A**) Age-dependent expression levels of Hap1 and c-kit in the hippocampus of mice at different ages. Both proteins peak in their expression at the postnatal stage. (**B**) Hippocampal neuronal culture at DIV5 was immunostained with antibodies against Hap1 and c-kit. The two proteins are coexpressed in the majority of cultured hippocampal neurons. Scale bar: 10 μm. (**C**) Brain sections from P15 WT mouse were immunostained with antibodies to Hap1 and c-kit. Hap1 and c-kit are coexpressed in some neurons in the hippocampal DG. Lower panel represents magnified images of the boxed areas in the upper panel. Scale bars: 40 μm (upper panel), 10 μm (lower panel). (**D**) Plasmid construct expressing c-kit was transfected into Neuro2A cells that had been treated with either scramble or Hap1 siRNA. Protein stability of c-kit was then assessed by cycloheximide (CHX) chase followed by western blot (left panel). Quantification of 3 independent experiments is shown on the right panel. Hap1 knockdown markedly decreased the half-life of c-kit in Neuro2A cells. Error bars represent SEM. *p<0.05.

We next looked at the expression level of c-kit in the hippocampus at different ages and found that, very similar to Hap1, the c-kit expression level peaks at the postnatal stage (Fig 6A), which supports a role for both proteins in the regulation of postnatal neurogenesis. To see whether Hap1 directly promotes the c-kit level in hippocampal neurons, we first examined whether these two proteins are expressed in the same neurons. Therefore, we cultured primary hippocampal neurons from P1–P2 WT pups and co-stained these neurons at DIV5 with antibodies for Hap1 and c-kit. We saw that Hap1 and c-kit were coexpressed in most neurons in this in vitro system (Fig 6B). To examine their expressions in vivo, we also performed co-immunofluorescent staining of Hap1 and c-kit in P12 WT mouse brain sections and found that Hap1 and c-kit were partially coexpressed in the subgranular zone of the DG, a region where new neurons are born (Fig 6C). To examine which type of cells in the DG express c-kit, we stained WT mouse brain sections from P7–P15 with antibodies for c-kit and an array of cell type-specific markers. We found that during the early postnatal stage, c-kit was expressed in NPCs, immature neurons, and mature neurons, as there was co-immunostaining of c-kit with nestin, DCX, or NeuN in the same cells (S4A Fig). In mature neurons, c-kit expression was found mainly in GAD67-expressing GABAergic interneurons, but not in prox1-expressing granule cells (S4A Fig). We also saw a similar pattern of expression for Hap1 at this age (S4B Fig). Therefore, it is possible that Hap1 and c-kit may directly regulate the proliferation and/or differentiation of hippocampal NPCs at an early postnatal stage, or that they might be involved in GABAergic control of hippocampal neurogenesis, a phenomenon reported previously [58–61]. In 1-month old mouse hippocampus, however, c-kit expression was largely restricted in GAD67-expressing GABAergic interneurons (S4A Fig). Since Hap1 could not be detected in NPCs in adult rat hippocampus, and more than 60% of Hap1-immunoreactive cells express GABA [62], it is likely that Hap1 and c-kit may mediate GABAergic control of adult hippocampal neurogenesis, which could serve as a buffer mechanism against stress-induced behavioral changes. Hap1 is known to stabilize internalized membrane receptors via its trafficking function between endosomes and lysosomes [29, 31, 32, 63–65]. To verify whether Hap1 directly regulates c-kit levels intracellularly, we measured the stability of transfected c-kit in Neuro2A cells. We found that knocking down endogenous Hap1 expression via siRNA substantially diminished the stability of c-kit (Fig 6D), indicating that direct intracellular regulation of c-kit levels by Hap1 is at least part of the mechanism by which Hap1 stabilizes c-kit.

### Overexpression of c-kit in the Hippocampus Rescues the Neurogenesis Defect and Adult Depressive-Like Behavior in *camk2a-Hap1* KO Mice

**Fig 7:**
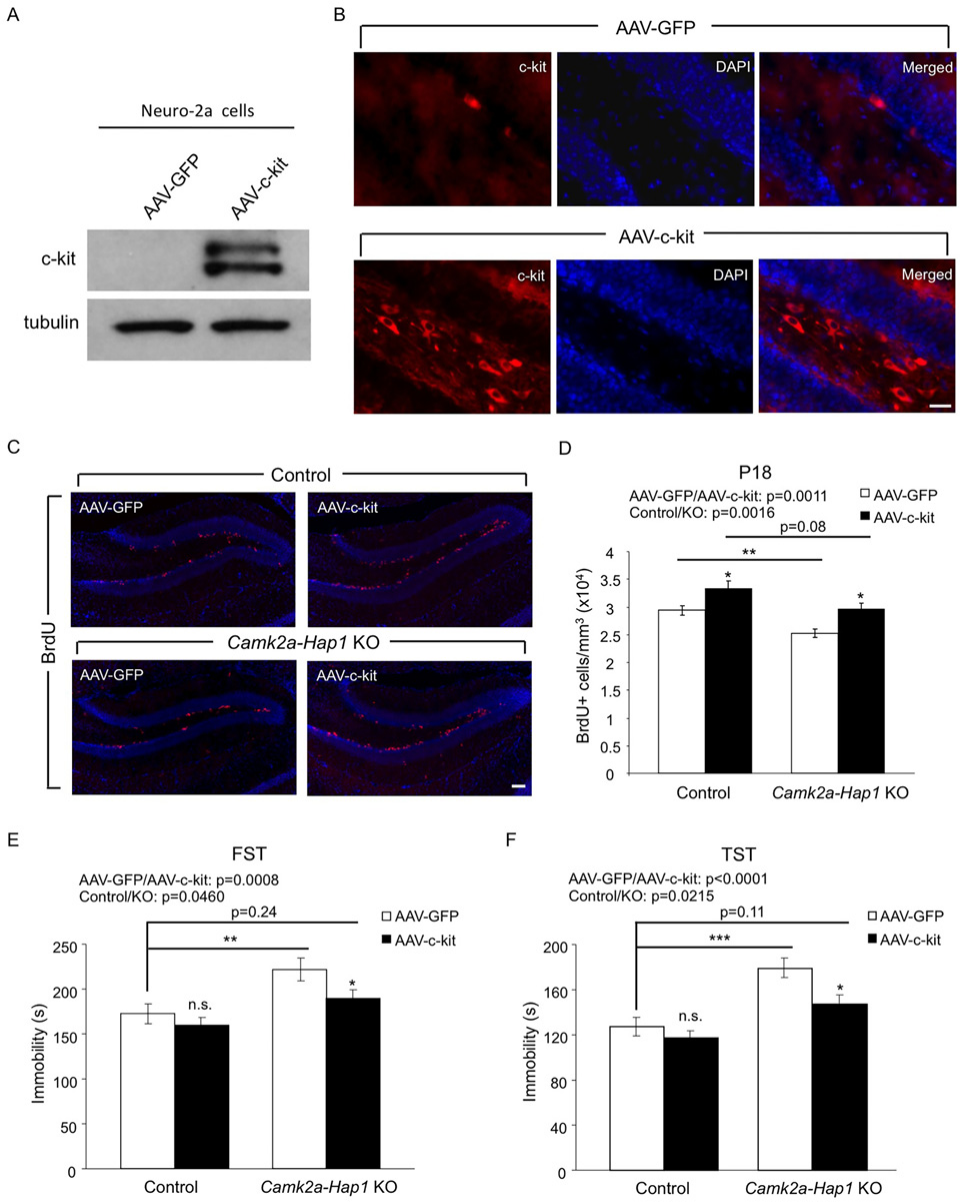
Overexpression of c-kit rescues postnatal neurogenesis defect and adult depressive-like phenotype in *camk2a-Hap1* KO mice. (**A**) Neuro-2A cells were infected with AAV-c-kit or control virus for 72 hours, and the expression of c-kit was analyzed by western blot. The top band may be the precursor of c-kit, which is not present in the brain lysate. (**B**) AAV-c-kit was stereotaxically injected into P3 mouse hippocampus. Two weeks later, the expression of c-kit in the hippocampus was examined by immunostaining, which showed robust expression of c-kit in the hilus and subgranular zone of the DG. AAV-GFP injection served as a control. (**C-D**) C*amk2a-Hap1* KO and control mice at P3 were injected with AAV-c-kit or control GFP virus into the hippocampus. BrdU was i.p. injected into these mice at P17, and 24 hours later, BrdU staining (C) and stereological quantification of BrdU+ cells (D, n = 4–6 per group) were performed, which revealed enhanced hippocampal neurogenesis by the overexpression of c-kit. (**E-F**) 2–3 months old *Camk2a-Hap1* KO and control mice, which were injected with AAV-c-kit or control virus into the hippocampus at P3, were subjected to the FST (E) and TST (F). Both tests demonstrated a rescue effect of c-kit on adult depressive-like behavior in *camk2a-Hap1* KO mice. n = 10–20 per group. All error bars represent SEM. n.s., not significant, *p<0.05, **p<0.01, ***p<0.001.

We next asked whether increasing the expression of c-kit in the hippocampus of *camk2a-Hap1* KO mice could rescue the neurogenesis defect and adult depressive-like behavior displayed by these mice. To this end, we designed adeno-associated virus (AAV) expressing c-kit under the synapsin-1 promoter and validated the successful infection of the virus and expression of c-kit both in Neuro-2a cells (Fig 7A) and mouse hippocampus, which was stereotaxically injected with AAV-c-kit at P3 (Fig 7B). The overexpression of c-kit in the hippocampus resulted in increased BrdU incorporation at P18 for both *camk2a-Hap1* KO and control mice (Fig 7C and 7D), suggesting that postnatal hippocampal neurogenesis can be modulated by altering the c-kit level. Furthermore, behavioral examination using the FST (Fig 7E) and TST (Fig 7F) showed that P3 AAV-c-kit injection in the hippocampus had a significant effect on mouse depressive-like behavior compared with control virus. Although c-kit overexpression slightly reduced the immobility of control mice in both tests, this overexpression in the hippocampus was able to significantly improve the performance of camk2a-Hap1 KO mice to a greater extent, thus partially ameliorating the adult depressive-like phenotype in these mice. As the virus directed the expression of c-kit in the hippocampus, the improved behavioral phenotype is very likely a specific result of the enhanced hippocampal neurogenesis by c-kit overexpression. Taken together, these results indicate that suppressed c-kit expression indeed accounts for both the neurogenesis defect and adult depressive behavior in mice lacking Hap1, and upregulation of c-kit or Hap1 levels could be considered as therapeutic options.

## Discussion

Since its identification as the first known interacting protein of huntingtin, Hap1 has been investigated in a number of studies for its role in both physiological and disease contexts. It has become evident that the neuronal expression of Hap1 is essential for early postnatal survival of mice, as germline *Hap1* KO results in an early postnatal lethal phenotype [30, 66]; however, whether Hap1 depletion induced at different ages has differential effects on adult animal behaviors has gone unexplored. Using an inducible *Hap1* KO mouse model, we were able to demonstrate that early postnatal Hap1 depletion leads to reduced neurogenesis in the hippocampus and depressive-like behavior in adult mice. Our studies thus demonstrate for the first time that genetic induction of defective postnatal neurogenesis can lead to adult depressive-like behavior.

Depression is a major mental disorder that affects hundreds of millions of people worldwide [67]. Due to its wide spectrum of symptoms, many genetic and environmental factors may trigger the onset of a depressive episode. These episodes are normally observed in adults, but are also seen in childhood and adolescence, although the latter cases are more difficult to diagnose and often go overlooked [68]. However, the importance of early life experience to adult depression is accentuated by the fact that, in the most severe cases of adult depression, some form of abuse was experienced in childhood [69]. It is therefore apparent that environmental factors in early life can contribute significantly to adult depression; whether genetic mutations also play a role in predisposing people with unpleasant early life experiences or even without such experiences to depression remains largely unknown. Our results using induced *Hap1* KO mice show that early loss of a gene involved in neurogenesis can indeed contribute to the etiology of depression, suggesting that early genetic diagnosis could possibly help with prediction of and early intervention for depression, or likely other forms of adult-onset mental disorders.

Hippocampal neurogenesis has emerged as an appealing theory to explain depression; however, a causal relationship between hippocampal neurogenesis and depression has not been established [70, 71]. Because neurogenesis happens more dynamically in the early postnatal brain than adult brain and since Hap1 regulates postnatal hypothalamic neurogenesis [32], we hypothesized that Hap1 may also regulate postnatal hippocampal neurogenesis, and the reduction of which could contribute to adult depressive behavior. We found that *Hap1* deletion at P1 significantly reduced hippocampal neurogenesis at the early postnatal stage and caused depressive-like behavior in adults. Since these P1 KO mice also showed severe growth retardation and reduced survival, we used *camk2a-Hap1* KO mice as another model for the study, which deletes *Hap1* mainly in the cortex and hippocampus from early postnatal life. These mice could survive and grow normally, yet exhibited a postnatal neurogenesis defect as well as adult depressive behavior, suggesting that reduced postnatal neurogenesis is more likely to be a cause of the depressive phenotype. In addition, we also found that loss of Hap1 reduces the expression of c-kit, which is present in progenitor cells for hippocampal neurogenesis. It should be pointed out that in conditional *Hap1* KO mice, Hap1 depletion, which is mediated by Cre under the control of *camk2a* promoter, is not restricted to the hippocampus. To explore whether Hap1 depletion in the postnatal hippocampus plays a pivotal role in adulthood depression, we used stereotaxic injection to overexpress c-kit in P3 mouse hippocampus and found that this overexpression can augment hippocampal neurogenesis and mitigate the depressive phenotype caused by the loss of Hap1, further supporting a causal role of reduced postnatal hippocampal neurogenesis in adult depression in our Hap1-deficient mice. All these findings provide evidence of a new role for Hap1 in adult depressive behavior.

Our earlier study suggested that *Hap1* is an essential gene for postnatal survival, but might be dispensable for adults [32]. However, mice used for those experiments were raised in standard housing conditions, which are far different from all the stress and threats animals would experience in the wild. In our case, although late postnatal or adult Hap1 depletion did not lead to overt phenotypes, its expression could be needed under certain stress conditions. Despite a lack of depressive phenotype caused by ablation of adult hippocampal neurogenesis, stress is found to down-regulate adult hippocampal neurogenesis, and adult-born hippocampal neurons are required for the regulation of the hypothalamic–pituitary–adrenal (HPA) axis in buffering stress-induced depressive behaviors [14, 21, 72]. Thus, an investigation of whether adult Hap1 expression influences hippocampal neurogenesis and depressive behavior under stressful conditions was of great interest to us. We found that 7-day repeated restraint stress was able to diminish adult hippocampal neurogenesis in P21 KO mice in which Hap1 depletion occurred after the postnatal period. This finding suggests that Hap1 expression may be required for adult hippocampal neurogenesis under stress and that its loss at least increases susceptibility to stressors in adulthood.

Since postnatal neurogenesis is necessary for the maturation of the central nervous system after birth, its reduction might affect the connectivity of certain critical neural circuitries, rendering animals susceptible to depression later in their lives. It is also possible that adult-expressed Hap1 plays an important role in maintaining a proper level of hippocampal neurogenesis and thus the hippocampal neural circuitry, which is needed for animals to buffer against stress-induced behavioral changes. Hap1 is known to interact with a number of endocytic receptors in a way that their degradation is inhibited and levels are stabilized [29–32, 63–65]. These receptors could be important for neurogenesis or other neuronal functions that are involved in maintaining and regulating hippocampal connectivity and circuitry in response to environmental stress. We conducted mass spectrometry analysis and found that c-kit, a protein that has been suggested to play a role in hippocampal neurogenesis and synaptic potentiation, as well as hippocampal-dependent learning and memory [40, 73, 74], was downregulated in *Hap1* KO hippocampus. Since c-kit also undergoes ligand-induced internalization [75, 76], it is likely that Hap1 functions to stabilize endocytic c-kit as it does to those other receptors. Moreover, Hap1 and c-kit both peak their expression levels in the hippocampus at early postnatal stage, and show a continued decrease in expression with age. Although this alteration is in line with the age-dependent decline in neurogenesis, it remains to be investigated whether increasing Hap1 expression or its mediated signaling, e.g., SCF/c-kit pathway in the adult brain can be beneficial to adult neurogenesis and reducing aging-related phenotypes [77, 78]. We found that Hap1 and c-kit are coexpressed in NPCs, immature neurons, and GABAergic interneurons in the DG during early postnatal life, though they become more restricted in interneurons later in life. Such different distributions could account for the differential roles of Hap1 in postnatal and adult neurogenesis and associated behavioral phenotypes. It is also possible that during the postnatal stage, Hap1 and c-kit regulate the proliferation and differentiation of a subpopulation of DG NPCs into interneurons, and once differentiated, they remain expressed in these interneurons, which can also regulate neurogenesis as reported previously [58–60]. As their expressions decrease significantly with age, Hap1 and c-kit might become part of the cellular machinery in response to stress or injuries as supported by the previous finding on c-kit [41]. The differential roles of Hap1 in postnatal and adult life are not unexpected as Hap1 is a multifaceted protein that interacts with different partners. Its association with other proteins and the resulting functions may be cell-type dependent and also depend on posttranslational modulations that can be cell-type and age-dependent. Whether Hap1 regulates c-kit function differently during postnatal and adult life requires further investigations. The neurogenesis and behavioral rescue in *camk2a-Hap1* KO mice via c-kit overexpression in the hippocampus suggests that c-kit-mediated signaling pathways are important for postnatal hippocampal neurogenesis and adult depressive behavior.

In conclusion, we have demonstrated a novel role for Hap1 in the regulation of postnatal neurogenesis and adult depressive-like behavior and provided the first genetic model that relates postnatal neurogenesis to adult depression. Our findings may help us better understand the mechanisms of depression, as well as identify potential therapeutic interventions.

## Materials and Methods

### Ethics Statement

All animal studies were performed in compliance with IACUC (Institutional Animal Care and Use Committee) at Emory University.

### Reagents and Antibodies

Dulbecco’s modified Eagle’s medium (DMEM), Neurobasal-A, B27, GlutaMAX-1, D-Hank’s, and fetal calf serum (FCS) were obtained from Life Technologies. Trypsin, poly-D-lysine, BSA, BrdU, imipramine, cycloheximide were all from Sigma. Cell culture dishes, coverslips, plates, and flasks were purchased from Corning and Nunc, Inc. Guinea pig antibody to Hap1 was generated in our laboratory [28, 30]. Rabbit anti-c-kit and phosphorylated-c-kit (Cell Signaling), rabbit anti-NeuN, mouse anti-NeuN, GFAP, nestin, GAD67, prox1 (all from Millipore), goat anti-DCX and guinea pig anti-DCX (Santa Cruz and Millipore), rat anti-BrdU (Accurate Chemical), rabbit anti-Ki67 (Thermal), mouse anti-calretinin (BD Transduction Laboratories), mouse anti-tubulin (Sigma) were used for western blot and immunofluorescent staining. Dilutions of the primary antibodies used can be found in S1 Table. HRP-tagged or fluorescent secondary antibodies were obtained from Jackson ImmunoResearch Laboratories.

### Animals

Mice were housed in the Division of Animal Resources at Emory University on a 12-h light (7 am-7 pm)/dark (7 pm-7 am) cycle. All procedures and husbandry were in accordance with the NIH Guide for the Care and Use of Laboratory Animals. Generation of germline *Hap1* KO mice (C57BL/6/black Swiss), floxed-*Hap1* mice (C57BL/6/SV129), and TM-inducible Cre-ER (C57BL/6/CBA)/floxed-*Hap1* mice was described in our previous studies [30, 32, 79]. Transgenic mice expressing Cre under the control of the mouse *calcium/calmodulin-dependent protein kinase II alpha* (*camk2a*) promoter (C57BL/6/BALB/C) were kindly provided by Dr. Stephen Warren (Emory University). *Camk2a-Hap1* KO mice were generated by crossing the floxed-*Hap1* mice with *camk2a*-Cre transgenic mice. Control mice were floxed-*Hap1* mice without transgenic Cre, and were all littermates of the KO mice.

### Western Blot Analysis

Mouse brain tissues or harvested cells were lysed in ice-cold RIPA buffer (50 mM Tris pH 8.0, 150 mM NaCl, 1 mM EDTA pH 8.0, 1 mM EGTA pH 8.0, 0.1% SDS, 0.5% DOC, and 1% Triton X-100) containing Halt protease inhibitor cocktail (Thermol Scientific) and phosphatase inhibitors. The lysates were incubated on ice for 30 min, sonicated, and centrifuged at top speed for 10 min. The supernatants were subjected to SDS-PAGE. The proteins on the gel were transferred to a nitrocellulose membrane, which was then blocked with 5% milk/PBS for 1 h at room temperature. The blot was incubated with primary antibodies in 3% BSA/PBS overnight at 4°C. After 3 washes in PBS, the blot was incubated with HRP-conjugated secondary antibodies in 5% milk/PBS for 1 h at room temperature. After 3 washes in PBS, ECL Prime (GE Healthcare) was then used to detect immunoreactive bands on the blot. A detailed list of the primary antibodies used can be found in S1 Table.

### Immunofluorescence Microscopy

For immunofluorescent staining of cultured neurons, neurons were washed once with PBS, and fixed with 4% paraformaldehyde (PFA) for 10 min. Fixed cells were washed 3 times with PBS, and then blocked with 3% BSA + 5% normal donkey serum/PBST (0.2% Triton X-100 in PBS) for 1 h at room temperature. Primary antibodies were diluted in blocking buffer and incubated with the cells at 4°C overnight followed by 3 washes with PBS and incubation with fluorescent secondary antibodies and nuclear dye. After 3 washes, the cells were ready for examination using a Zeiss (Axiovert 200M, Germany) microscope with a digital camera (Orca-100; Hamamatsu Photonics, Bridgewater, NJ) and the Openlab software (Improvision, Lexington, MA). Immunofluorescent staining of brain sections was performed as described previously [29, 38]. Briefly, mice were deeply anesthetized, perfused with saline followed by 4% PFA fixation. Brains were postfixed overnight in the same fixative, and switched to 30% sucrose at 4°C. After completely sunk, brains were sectioned at 15 μm (40 μm for BrdU staining) with a cryostat at −19°C and mounted onto gelatin-coated slides. The tissues on the slides were washed, blocked, and immunostained with antibodies using the same method described above for cultured cells. For BrdU immunostaining, sections were first treated with 2 N HCl for 30 min at 37°C and then neutralized with 0.1 M sodium borate (pH 8.5) for 15 min at room temperature. A detailed list of the primary antibodies used can be found in S1 Table.

### TM Induction in Mice

TM induction in mice was performed as described previously [32]. Briefly, TM (Sigma T5648) was dissolved in ethanol at 20 mg/ml and stored at -20°C before use. To induce *Hap1* depletion, a calculated amount of TM was mixed with corn oil, and ethanol was removed by a vacuum centrifuge. 1.1 mg or 2.2 mg TM per 40 g body weight was used to inject P1 (subcutaneous) or P10 (i.p.) mouse pups for 3 consecutive days. For mice at P15 or older, 4 mg TM per 40 g body weight was used for i.p. injection for 5 consecutive days. Both TM-inducible Cre-ER/floxed-*Hap1* mice or *camk2a-Hap1* KO mice and their control littermates were given TM injections at the same time.

### BrdU Incorporation Assay

For BrdU injection into *Hap1* P1 KO mice and controls, mice at P6 were i.p. injected with 50 mg/kg body weight BrdU. The animals were perfused and fixed 24 hours later for the analysis of NPC proliferation, or 4 weeks later for the analysis of neural differentiation. For BrdU injection into *camk2a-Hap1* KO or P21 KO mice and controls, mice at P33 (or P17 for AAV-c-kit rescue experiment) were i.p. injected with 50mg/kg body weight BrdU. Twenty four hours later, the mice were perfused and fixed for analyzing the number of proliferating cells.

### Stereology and Quantification

Stereological cell counting and quantification were performed as described in our previous study [32]. Briefly, to quantify BrdU+ cells, the optical-fractionator method implemented in Stereo Investigator 9.03.2 (MicroBrightField, Magdeburg, Germany) was used. One-in-six 40-μm serial sections covering the entire hippocampal region were stained to visualize and quantify BrdU+ cells in the DG. A minimum of 3 mice from each genotype and 8 sections from each mouse brain were examined for comparisons. The volume of the DG and the total number of BrdU+ cells in the DG were calculated by Stereo Investigator software. The total number of BrdU+ cells was then divided by the volume to yield cell density presented as the number of BrdU+ cells per mm^3^. For estimation of the ratios of NeuN+/BrdU+ and GFAP+/BrdU+ cells among BrdU+ cells, sections were co-stained for BrdU and either NeuN or GFAP. Each BrdU+ cell counted was also examined for the presence of NeuN or GFAP labeling, and the double-positive cells were marked and quantified separately. The densities of the double-positive cells were divided by those of the total BrdU+ cells to yield the ratios. Counting of the cells was performed under the 40× lens in a Zeiss AX10 microscope.

### Forced Swim Test (FST)

In multiple experiments, conditional *Hap1* KO mice and their control littermates (age indicated in figure legends) were placed individually into a round opaque plastic cylinder (18 cm in height, 15 cm in diameter) filled with water (25°C) at a depth of 12 cm. Immobility time, defined as floating or the absence of active behaviors, such as swimming or struggling to escape, was measured. Slight movements of the feet and tail necessary to keep the head above water were excluded as mobility. Each mouse was measured for 6 min by a trained observer who was kept blind to the genotypes of the mice and drug treatment. No pretest training of mice was performed.

### Tail Suspension Test (TST)

As for FST, conditional *Hap1* KO mice and their control littermates (age indicated in figure legends) were used for TST in multiple experiments. The mice were suspended by taping the tail (~1 cm from tip of tail) to a horizontal bar at a height of 40 cm from the table surface for 6 min. The trial was conducted for a duration of 6 min, and the immobility time was recorded manually via stopwatch by a trained observer who was blind to the genotypes of the mice examined. Mice were considered immobile when they hung passively and motionlessly without escape-oriented behaviors.

### Rotarod Test

Motor activity was evaluated using Rotamex (Columbus Instruments). Two-month old *Hap1* P1 KO or *camk2a-Hap1* KO mice and their control littermates were trained on a rotating rod at a speed of 5 rpm for three 5-min trials on 3 consecutive days. Testing was performed on the fourth day. During the test, the rotating rod was gradually accelerated to 40 rpm over 5 min. Latency to fall from the rotarod was recorded in 3 trials, and the average of the 3 trials was used for each mouse.

### Imipramine Treatment

Tricyclic antidepressant imipramine (30 mg/kg, sigma) was freshly made in saline and i.p. injected into 4-month old *camk2a-Hap1 KO* or control mice 30 min before FST or TST. Saline injection was used as vehicle treatment.

### Restraint Stress

One-year old *Hap1* P21 KO and control mice were subjected to repeated restraint stress by placement for 4 h per day for 7 consecutive days in ventilated 50 ml conical tubes. After each stress session, mice were immediately returned to their home cages. Control mice were housed in separate cages from the stressed mice, and were deprived of food and water but otherwise untouched during each session. Twenty four hours after the final session, mice were evaluated by FST. For neurogenesis analysis, BrdU (100mg/kg body weight) was i.p. injected before the stress session on each of the last 3 days. Twenty four hours after the final session, mice were perfused and fixed, their brains were then sectioned for BrdU immunostaining.

### Locomotor Activity Assay

Locomotor activity was measured using an automated system (San Diego Instruments, La Jolla, CA) with photobeams that record ambulations (consecutive beam breaks). Two-month old *Hap1* P1 KO and control mice were individually placed in the chambers under 12-h light-dark cycle with free access to food and water. Mice were allowed 4 h to acclimate to the new environment before recording. Activities were recorded every 30 min for 24 h.

### Sucrose Preference Test

The sucrose preference test was conducted as previously described with modification [21]. Briefly, 2-month old *camk2a-Hap1* KO and control mice were individually housed with free access to food and two weighed bottles of liquid: one filled with water, the other with 1% sucrose solution. The positions of the two bottles were balanced across animals. After 3 days of acclimation, both bottles were removed and weighed at 12 pm, and then put back in reversed positions at 7 pm. The bottles were weighed again in 1 h for an acute test, and again on the next morning for an overnight test. Sucrose preference was calculated as (Δweight_sucrose_)/(Δweight_sucrose_ + Δweight_water_) × 100.

### Hippocampal Neuronal Cell Culture

Hippocampal dissection and neuronal cell culture were performed as previously described [80]. Briefly, hippocampi were dissected from P1 WT mice and placed in a sterile 35-mm petri dish containing ice-cold Hanks’ balanced salt solution (HBSS, Ca^2+^-and Mg^2+^-free), chopped into 1 mm^3^ pieces by microscissors, and digested with 0.125% (w/v) trypsin at 37°C for 25 min. The enzymatic activity was terminated by adding DNaseI (200 U/ml final concentration) and heat-inactivated FCS (20% final concentration) into the solution. The tissue was then dissociated by triturating through a fire-polished Pasteur pipette, spun down and washed twice with culture medium (Neurobasal-A supplemented with B27 and GlutaMAX-1). After resuspension in culture medium, 2 × 10^5^ cells per well were plated onto 6-well plates. Neurons that were cultured for 5 days *in vitro* (DIV) were fixed by 4% PFA and used for immunofluorescent staining.

### Preparation of Adenoviral Hap1 siRNA and AAV-c-kit

Adenoviral Hap1-specific and scramble siRNAs were prepared in our previous study [81]. Viral stocks were adjusted to 1X10^8^ viral particles/μl before use. Neuro2A cells were incubated with adenoviral Hap1 siRNA at a multiplicity of infection of 50. Twenty-four h after infection, the virus-containing medium was removed, and the c-kit plasmid was transfected for another 48 h before performing the protein stability assay. Mouse c-kit cDNA was subcloned into a pAAV-MCS vector (Cell Biolabs). The human synapsin-1 promoter sequence was inserted into the construct to replace the original promoter in the vector. AAV-c-kit (Serotype 9) was packaged and amplified by the viral vector core at Emory University. AAV-GFP (Serotype 9, SignaGen Laboratories) under the same promoter was used as a control.

### Stereotaxic Viral Injection

A *camk2a-Hap1* KO or control mouse pup at P3 was placed in a latex sleeve and immersed up to the neck in crushed ice and water (2–3°C) for 7–10 min. The pup was then placed on an ice pack (3–4°C) and stabilized on a platform while being injected with 0.5 μl of AAV-c-kit or AAV-GFP viral particles (1X10^12^ particles/ml) into each side of the hippocampus (1.5 mm lateral from the sagittal suture, 2 mm rostral to the lambda, and 2 mm below the skull) over 2 min from a 5-μl Hamilton syringe and 33-gauge needle. The needle was kept still for another 2 min before withdrawal. The surgical field was illuminated with fiber optic to minimize inadvertent and uncontrollable warming. The pup was then transferred into a clean cage placed on a heat pad (33°C) with nest for 30 min to recover from hypothermia before returning to the home cage. We used 4–6 pups per experimental group.

### Statistical Analysis

All data are expressed as mean ±SEM. The statistical significance was determined by two-tailed Student’s t-tests or two-way ANOVA followed when appropriate by post hoc t-tests using GraphPad Prism 5.0 software. A value of *p*<0.05 was considered statistically significant.

## Supporting Information

S1 Fig Depleted Hap1 protein expressions in mice that had *Hap1* gene deleted at different time points. Whole brain tissues from 3-month old *Hap1* P1, P10, P15, P21 KO mice, and brainstem (B.S.) from a 4-month old *Hap1* adult KO mouse that had *Hap1* deleted at 2 month of age were used. Controls were littermates that were also given TM. Substantial depletion of Hap1 can be found in all KO tissues.
(TIF)

S2 Fig Increased immobility in depression tests caused by loss of Hap1 is not due to impaired motor function, and can be rescued by acute antidepressant treatment. (**A**) Rotarod analysis of 2-month old *Hap1* P1 KO or *camk2a-Hap1* KO mice and their controls. The P1 KO mice showed an increased rotarod performance, whereas *camk2a-Hap1* KO mice did not differ from controls. n = 10–17 per genotype. (**B**) Imipramine (30mg/kg) was i.p. injected 30 min before FST and TST on 4-month old *camk2a-Hap1* KO mice and controls. n = 10–13 per group. All error bars represent SEM. n.s., not significant, **p<0.01, ***p<0.001.
(TIF)

S3 Fig Decreased level of c-kit in *Hap1* P1 KO, but not adult KO, mouse hippocampus. (**A**) c-kit protein levels were assessed in 1 month-old *Hap1* P1 KO, and 3 month-old adult KO mouse hippocampus by western blotting. TrkB level was also examined. (**B**) Quantification of the western blotting results represented in (A) was done with 3 or 4 samples from each genotype. Ratios were normalized to controls. Compared with controls, c-kit level was reduced in P1 KO, but not adult KO mouse hippocampus. TrkB level was not significantly changed in either of the KO mouse hippocampus.
(TIF)

S4 Fig Distribution of c-kit immunoreactive cells in the hippocampal DG at early and late postnatal stages. (**A**) Double immunofluorescent staining of c-kit with specific markers for NPCs (nestin), immature neurons (DCX), mature neurons (NeuN), GABAergic interneurons (GAD67), and DG granule cells (prox1) in P7–P15 and 1 month-old mouse hippocampal DG indicates that c-kit can be expressed in populations of NPCs, immature neurons, and GABAergic interneurons at the early postnatal stage. At 1 month of age, c-kit expression is largely restricted in GAD67+ GABAergic interneurons. Scale bars: 10 μm. (**B**) Double staining of P7–P15 mouse hippocampus with antibodies to Hap1, DCX, GAD67, and Prox1 also showing the expression of Hap1 in GAD67+ GABAergic interneurons. Scale bars: 10 μm.
(TIF)

S1 Table Information on the primary antibodies used in western blotting (WB) and immunofluorescent staining (IF). (DOCX)
