# Supplementary figures and images for: Postnatal Loss of Hap1 Reduces Hippocampal Neurogenesis and Causes Adult Depressive-Like Behavior in Mice
Source: PLoS Genet. 2015 Apr 15;11(4):e1005175. doi: 10.1371/journal.pgen.1005175 (PMC4398408; doi:10.1371/journal.pgen.1005175)

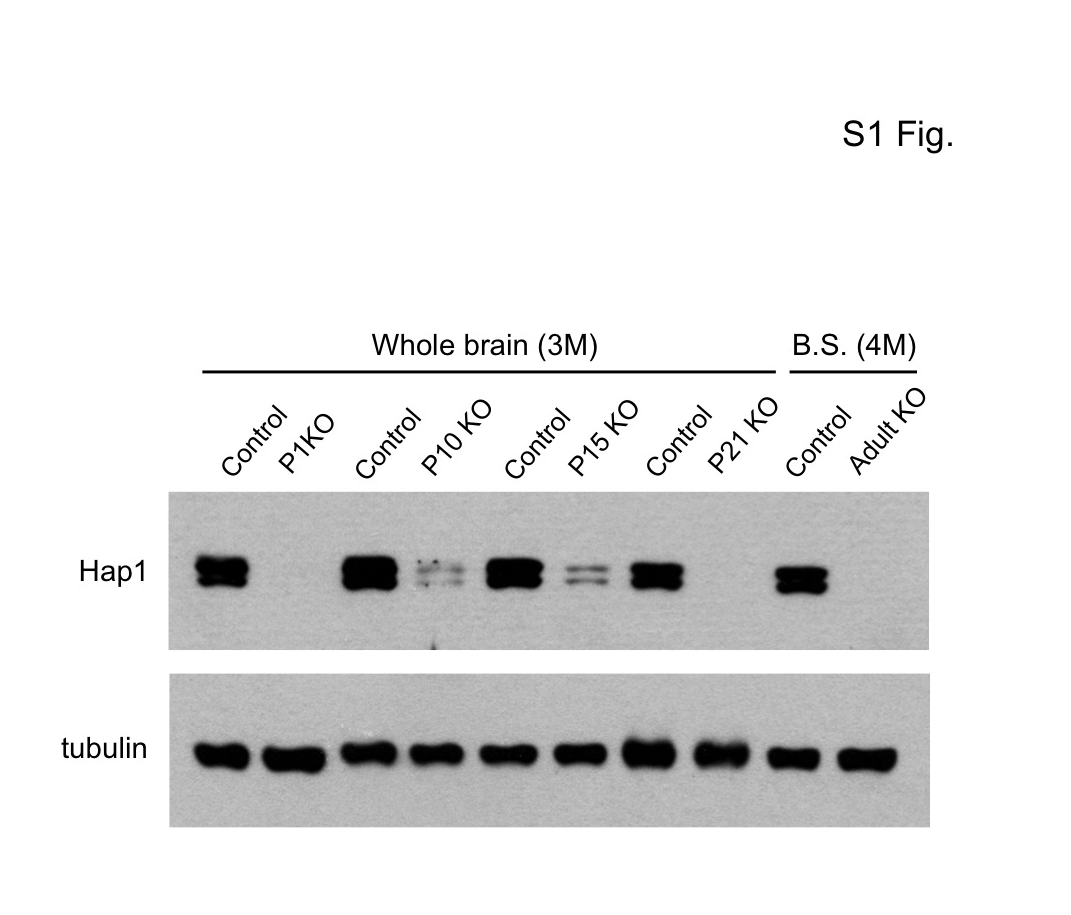

Supplement: S1 Fig — Whole brain tissues from 3-month old Hap1 P1, P10, P15, P21 KO mice, and brainstem (B.S.) from a 4-month old Hap1 adult KO mouse that had Hap1 deleted at 2 month of age were used. Controls were littermates that were also given TM. Substantial depletion of Hap1 can be found in all KO tissues. (TIF) [file pgen.1005175.s001.tif]

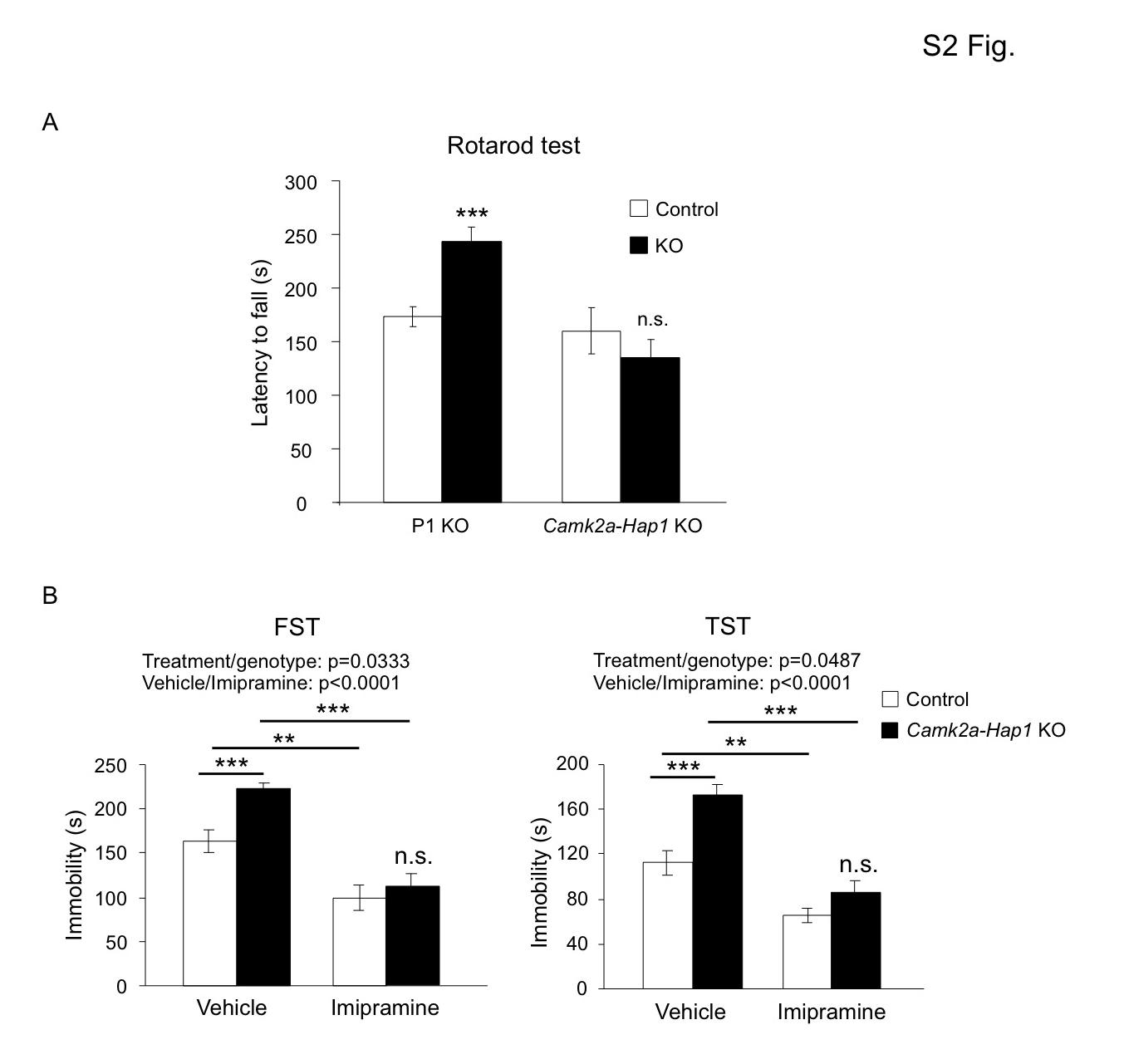

Supplement: S2 Fig — (A) Rotarod analysis of 2-month old Hap1 P1 KO or camk2a-Hap1 KO mice and their controls. The P1 KO mice showed an increased rotarod performance, whereas camk2a-Hap1 KO mice did not differ from controls. n = 10–17 per genotype. (B) Imipramine (30mg/kg) was i.p. injected 30 min before FST and TST on 4-month old camk2a-Hap1 KO mice and controls. n = 10–13 per group. All error bars represent SEM. n.s., not significant, **p<0.01, ***p<0.001. (TIF) [file pgen.1005175.s002.tif]

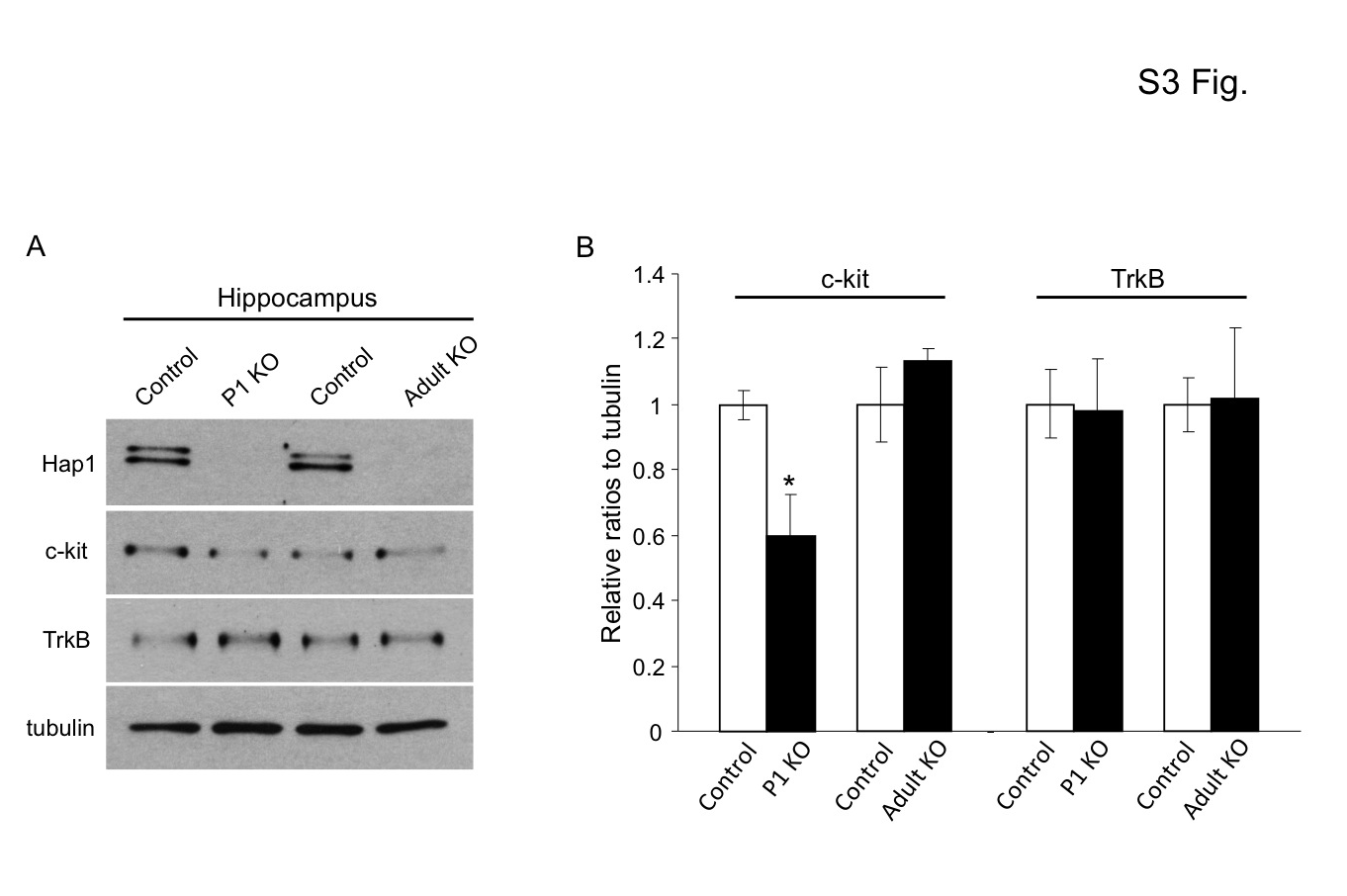

Supplement: S3 Fig — (A) c-kit protein levels were assessed in 1 month-old Hap1 P1 KO, and 3 month-old adult KO mouse hippocampus by western blotting. TrkB level was also examined. (B) Quantification of the western blotting results represented in (A) was done with 3 or 4 samples from each genotype. Ratios were normalized to controls. Compared with controls, c-kit level was reduced in P1 KO, but not adult KO mouse hippocampus. TrkB level was not significantly changed in either of the KO mouse hippocampus. (TIF) [file pgen.1005175.s003.tif]

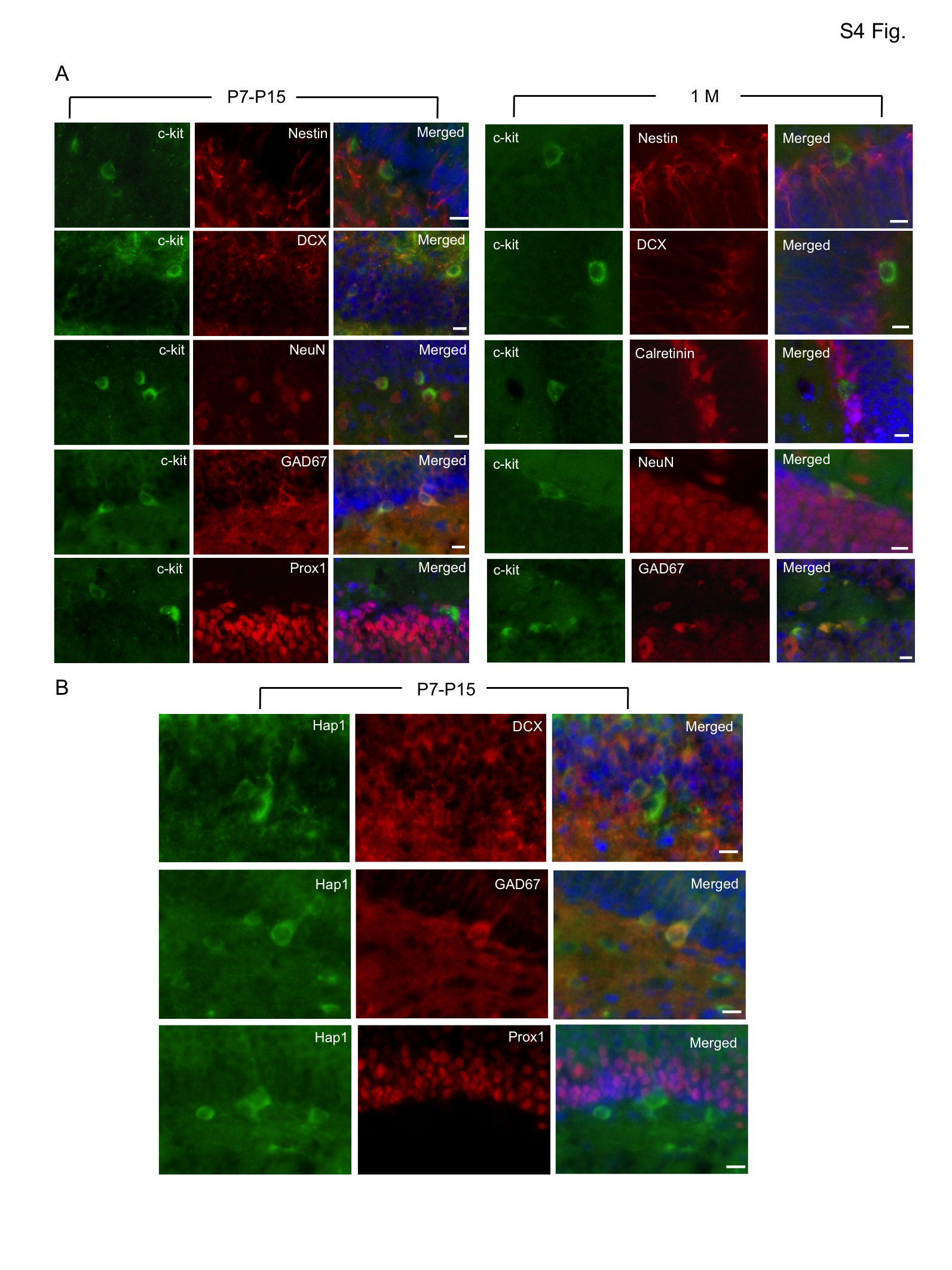

Supplement: S4 Fig — (A) Double immunofluorescent staining of c-kit with specific markers for NPCs (nestin), immature neurons (DCX), mature neurons (NeuN), GABAergic interneurons (GAD67), and DG granule cells (prox1) in P7–P15 and 1 month-old mouse hippocampal DG indicates that c-kit can be expressed in populations of NPCs, immature neurons, and GABAergic interneurons at the early postnatal stage. At 1 month of age, c-kit expression is largely restricted in GAD67+ GABAergic interneurons. Scale bars: 10 μm. (B) Double staining of P7–P15 mouse hippocampus with antibodies to Hap1, DCX, GAD67, and Prox1 also showing the expression of Hap1 in GAD67+ GABAergic interneurons. Scale bars: 10 μm. (TIF) [file pgen.1005175.s004.tif]
