# Supplementary material for: Postnatal Loss of Hap1 Reduces Hippocampal Neurogenesis and Causes Adult Depressive-Like Behavior in Mice
Source: PLoS Genet. 2015 Apr 15;11(4):e1005175. doi: 10.1371/journal.pgen.1005175 (PMC4398408; doi:10.1371/journal.pgen.1005175)
Supplement: S1 Table — (DOCX) [file pgen.1005175.s005.docx]

**Supplemental Table 1.** Information on the primary antibodies used in western blotting (WB) and immunofluorescent staining (IF).

| **Antibody** | **Company** | **Cat. No.** | **Host** | **Dilution**  **WB** | **Dilution**  **IF** |
| --- | --- | --- | --- | --- | --- |
| c-kit | Cell Signaling | D13A2 | Rabbit | 1:1,000 | 1:400 |
| Phospho-c-kit | Cell Signaling | #3391 | Rabbit | 1:500 |  |
| NeuN | Millipore | ABN78 | Rabbit | 1:20,000 |  |
| NeuN | Millipore | MAB377 | Mouse |  | 1:500 |
| GFAP | Millipore | MAB360 | Mouse |  | 1:800 |
| Nestin | Millipore | MAB353 | Mouse |  | 1:200 |
| GAD67 | Millipore | MAB5406 | Mouse |  | 1:200 |
| Prox1 | Millipore | MAB5654 | Mouse |  | 1:200 |
| DCX | Millipore | AB2253 | Guinea Pig | 1:10,000 | 1:2,000 |
| DCX | Santa Cruz | C-18 | Goat |  | 1:500 |
| BrdU | Accurate Chemical | OBT0030 | Rat |  | 1:200 |
| γ-Tubulin | Sigma | T6557 | Mouse | 1:20,000 |  |
| Ki67 | Thermal | Sp6 | Rabbit | 1:200 |  |
| Calretinin | BD Transduction Laboratories | 610908 | Mouse |  | 1:2,000 |
| Hap1 |  | EM77 | Guinea Pig | 1:10,000 | 1:500 |
